# Comparative metagenomics analysis reveals how the diet shapes the gut microbiota in several small mammals

**DOI:** 10.1002/ece3.8470

**Published:** 2022-01-15

**Authors:** Fengjun Li, Shengzhi Yang, Linwan Zhang, Lu Qiao, Lei Wang, Song He, Jian Li, Nan Yang, Bisong Yue, Chuang Zhou

**Affiliations:** ^1^ Key Laboratory of Bioresources and Ecoenvironment (Ministry of Education) College of Life Sciences Sichuan University Chengdu China; ^2^ Laojunshan National Nature Reserve Sichuan Province Pingshan China; ^3^ Institute of Qinghai‐Tibetan Plateau Southwest Minzu University Chengdu China

**Keywords:** diet, gut microbiome, high‐throughput sequencing, metagenomics, Muridae, Soricidea

## Abstract

The gut microbiomes of the host are large and complex communities, which helps to maintain homeostasis, improves digestive efficiency, and promotes the development of the immune system. The small mammals distributed in Sichuan Province are the most popular species for biodiversity research in Southwest China. However, the effects of different diets on the structure and function of the gut microbial community of these small mammals are poorly understood. In this study, whole‐metagenome shotgun sequencing has been used to analyze the composition and functional structures of the gut microbiota of seven small mammals in Laojunshan National Nature Reserve, Sichuan Province, China. Taxonomic classification revealed that the most abundant phyla in the gut of seven small mammals were Bacteroides, Proteobacteria, and Firmicutes. Moreover, *Hafni*a, *Lactobacillus*, and *Yersinia* were the most abundant genus in the gut microbiomes of these seven species. At the functional level, we annotated a series of KEGG functional pathways, six Cazy categories, and 46,163 AROs in the gut microbiomes of the seven species. Comparative analysis found that the difference in the gut microbiomes between the Soricidea and Muridae concentrated on the increase in the F/B (Firmicutes/Bacteroides) ratio in the Soricidea group, probably driven by the high‐fat and ‐calorie digestive requirements due to their insectivorous diet. The comparative functional profiling revealed that functions related to metabolism and carbohydrates were significantly more abundant in Muridae group, which may be attributed to their high carbohydrate digestion requirements caused by their herbivorous diet. These data suggested that different diets in the host may play an important role in shaping the gut microbiota, and lay the foundation for teasing apart the influences of heritable and environmental factors on the evolution of gut microbial communities.

## Graphical abstract

Our study illustrates the use of different diets to resolve potentially relevant host characteristics, providing nuanced insights into the manner by which gut microbes assort among host species.

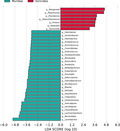

## 1 INTRODUCTION

Many studies have shown that the interaction between the gut microbial mutualisms, commensalisms, and pathogenicity is extremely critical to the host (Backhed et al., 2005; Kau et al., 2011). The gut microbiomes of small mammals have displayed the phylogenetic history of their hosts, indicating codiversification and evolutionary timescales between different small mammal species (Ji & Nielsen, 2015; Ochman et al., 2010). For the past 10 years, many studies on gut microbial community have been conducted on small mammals like desert woodrats (Kohl et al., 2015), wild wood mice (Maurice et al., 2015), the Western house mouse (Lorenc et al., 2014), plateau pika (Li, Li, et al., 2016; Li, Qu, et al., 2016), and the bank vole (Lavrinienko et al., 2018). These researches on small mammals were involved in the host intestinal peristalsis, nutrient absorption, energy metabolism, and immune homeostasis (He et al., 2018; O'Mahony et al., 2015).

The influences of host phylogeny and diet on gut microbiome structure and function have been widely explored. A comparison study from 60 species on gut microbiota displayed that the difference within species was less than that among species (Ley et al., 2008). Likewise, a comparison of 10 genetically distinct inbred mouse strains with sympatric distribution revealed that there was some convergence of gut microbiota between the strains, but each mouse strain retained a distinct microbiota (Campbell et al., 2012). In wild mice, the patterns of microbiota diversity were primarily explained by the geographical location of the mice, with weaker effects from the population structure of the mice and their genetic distance (Linnenbrink et al., 2013; Wang et al., 2014). When wild mice were transferred to the laboratory and maintained for a year, the diversity of the gut microbiota was relatively declining over time (Kohl et al., 2015; Wang et al., 2014), suggesting that short‐term and dramatic dietary interventions could alter the microbiota diversity quickly (Leeming et al., 2019). Diet is a key modifiable factor influencing the composition of the gut microbiota. Robust findings displayed that the gut microbiota responded rapidly to diet alteration appeared to be temporary when the dietary changes were permanent (Tebani & Bekri, 2019; Wu et al., 2011). Permanent alteration of the diet may induce new species and proliferate others, increasing the diversity and richness of taxa in gut microbiota (Linnenbrink et al., 2013). A diverse diet, and in particular, the number of different types of plant foods consumed (Johnson et al., 2019), has been associated with greater microbial diversity thought to provide an increased variety of substrates for numerous taxa proliferation (Heiman & Greenway, 2016; McDonald et al., 2018). Animal fat and protein predominant diets have been clearly associated with specific changes in gut microbial composition when compared to plant‐based diets (Johnson et al., 2019). For example, changes in the Firmicutes: Bacteroidetes ratio have been reported in individuals who lost weight, whether they were consuming low‐calorie, fat‐ or carbohydrate‐restricted diets (Ley et al., 2006).

The mammalian gut microbiota is a collection of all host gut‐related microorganisms, and it is a complex and diverse ecosystem that is obtained through vertical transmission and environmental exposure (Norman et al., 2014; Stappenbeck & Virgin, 2016). In small mammals, environmental exposure may cause the occurrence of antimicrobial resistance (AMR), referring to the physical or biochemical ability of microorganisms (usually pathogenic) to develop ineffective antibacterial agents, which is increasingly prevalent (Butaye et al., 2015). The capacity of small mammals to act as reservoirs and vectors of AMR indicates their potential use as sentinels of AMR occurrence, transmission, and potential human health risks. Sentinels or bioindicators are organisms with the potential to be used as an early warning system for human health risks (Gwenzi et al., 2021). A few studies showed that small animals, including mice, voles, and insectivores, are effectively used as sentinels of antimicrobial‐resistant microorganisms and their antibiotic resistance genes (ARGs) (Furness et al., 2017; Kmet et al., 2018).

Laojunshan National Nature Reserve in Sichuan Province is located on the southern edge of the Sichuan Basin connected to the hills of Southern Sichuan Basin and the Yunnan Plateau. Previous research studies on gut microbiomes of small mammals in Sichuan Province have been conducted based on the analysis of the composition and function of the gut microbiota, which was involved in habitat and seasonal changes (Maurice et al., 2015; Tang et al., 2021). As the sampled populations in these studies are ecologically, geographically, and seasonally distinct, it is unclear of the key functional bacteria and their mechanism in the gut and the impact of different diets on sympatric small mammals. Therefore, species with different diets in Laojunshan National Nature Reserve can provide reference for the study of the gut microbiomes of small mammals under sympatric distribution.

Due to the elusive predatory behavior and various habits, it is difficult to accurately and effectively evaluate the gut microbes of wild animals (Dettki et al., 2014; Rosshart et al., 2017). It is nearly impossible to identify gut microbe with high taxonomic resolution by using traditional methods such as microbial plate method, microscopic analysis, and phospholipid fatty acid analysis (Tannock, 2000; Wang et al., 2017; Xu et al., 2009). With the development of high‐throughput sequencing, many targeted amplicon sequencing technologies have been widely adopted for the study of microbiomes, including 16S rRNA gene and shotgun metagenomics (Kau et al., 2011; Li, Li, et al., 2016; Maurice et al., 2015; Wang et al., 2017). The main difference in these two methods is that 16S rRNA sequencing data cannot provide further insight into the functional capabilities of these gut microbiota, while the metagenomics can analyze the function of the gut microbes (Ji & Nielsen, 2015; Lesker et al., 2020). There is limited information on the functional diversity of gut microbiome in wild small mammals. Therefore, accurate identification of gut microbiota is a prerequisite to fully understand the feeding ecology of a species.

In this study, we used whole‐metagenome shotgun sequencing at the Illumina high‐throughput sequencing platform NovaSeq 6000 to profile the gut microbiomes inhabiting the digestive system of seven sympatric small mammals and identify the functional attributes encoded in the gut microbiomes. We chose five Muridae species: Eurasian Harvest Mouse (*Micromys minutus*), Confucian Niviventer (*Niviventer confucianus*), Indochinese Arboreal Niviventer (*Niviventer fulvescens*), Oriental House Rat (*Rattus tanezumi*), and *Apodemus nigrus*. These five species have been regarded as herbivorous animals (Du et al., 2017; Parr et al., 2014; Romer, 1966) with a dental system adapted to gnawing and grinding vegetable food (Butet et al., 2011; Hansson, 1985; Luckett & Hartenberger, 1985). Another two species were carnivorous Soricidea species: Indochinese short‐tailed shrew (*Blarinella griselda*) and the Chinese mole shrew (*Anourosorex squamipes*). Both species have been revealed to be insectivore animals, and they belong to carnivorous animals, which live on diverse invertebrates with a preponderance of earthworms (Churchfield et al., 2012; Pascual & Ascencao, 2000; Peng et al., 2018; Tang et al., 2021). This study can help better understand the composition and function of the gut microbiomes of the wild small mammals and lay the foundation for future research on how different feeding strategies affect the interaction of the small mammals and gut microbes.

## 2 MATERIALS AND METHODS

### 2.1 Ethics approval

The research complied with the protocols established by the China Wildlife Conservation Association and the legal requirements of China. The research protocol was reviewed and approved by the Ethical Committee of Sichuan University.

### 2.2 Sample collection and gut content samples

The samples of small mammals were collected from June 20 to June 26, 2020, using the snap‐trap cage at night in Laojunshan National Nature Reserve, Sichuan Province, Southwest China. We selected the samples to be analyzed according to the following criteria. First, samples were collected from family Muridae and Soricidea at the same site in 1 week. Second, each species has at least three samples. Third, the samples had complete stomach, intestine, and anal canal. A total of seven species, and three sample replicates for each species were analyzed in this study (Table S1). The sampling sites occupied a range of elevations from 1460 to 1861 m, longitudes from 103.99°E to 104.22°E, and latitudes from 28.60°N to 28.70°N. The internal organs and digestive tract of samples were collected and placed in 100 ml sterilized plastic bottles and sealed with pure alcohol. In addition, part of the muscle tissue is collected in a 2 ml EP tube with pure alcohol. The fur specimens were labeled and stored at 10% formalin solution. As soon as animal specimens were collected, the samples were immediately transported to the College of Life Sciences, Sichuan University, and stored at −80°C. The entire sampling process was aseptic. The luminal gut contents were collected at a super clean bench. Due to the dehydration effect of pure alcohol, we directly dissected the stomach and intestines of the samples, collected their contents, and stored them in pure alcohol.

### 2.3 DNA extraction, library preparation, and metagenomics sequencing

Metagenomic DNA was isolated from approximately 1 g of gut content sample with the Magnetic Bead Method Soil and Fecal Genomic DNA Extraction Kit (DP712, TIANGEN Biotech, Beijing, China), following the manufacturer's instruction. DNA concentration and quality were assessed by three methods: agarose gel electrophoresis analyzed the purity and integrity of DNA; Nanodrop detected the purity of DNA (OD 260/280 ratio); and Qubit 2.0 quantified DNA concentration. DNA samples were randomly interrupted using a Covaris ultrasonic disruptor, and the entire library preparation was constructed through end repair, A‐tailing, sequencing adapters, purification, and PCR amplification. We then used Qubit 2.0 for preliminary quantification. To ensure the quality of the library, we used Agilent 2100 to detect the inserts in the library and the Q‐PCR method to accurately quantify the effective concentration of the library. Different qualified libraries were pooled to flowcell according to the effective concentration and target data volume requirements. After the cBOT was clustered, the Illumina high‐throughput sequencing platform NovaSeq 6000 was used for sequencing. The details of data analysis are attached in Table S2.

### 2.4 Quality control and genome assembly

To obtain the clean data for subsequent analysis, the raw data from the Illumina NovaSeq sequencing platform were processed by removing the reads that contain low‐quality bases more than a certain percentage (default is 40 bp); the reads in which the N base reached a certain percentage (default length of 10 bp); and the reads whose overlap with the adapter exceeded a certain threshold (default is 15 bp). Because the sequencing data had host contamination, we compared with the reference genome sequence from NCBI (https://www.ncbi.nlm.nih.gov/) (Muridae: GCF_011064425.1; Soricidea: GCF_000181275.1) to filter out the possible source of the host reads. We used MEGAHIT to assemble the metagenomics data (Li et al., 2015) (https://github.com/voutcn/megahit). Then, the assembled sequences were further analyzed by QUAST producing genomes with contigs of 300 bp or more, which was used for further gene prediction and annotation (Gurevich et al., 2013).

### 2.5 Gene prediction and gene abundance analysis

The gene prediction was performed with the help of Prodigal (version 2.60; Hyatt et al., 2000). All genes predicted to have 95% sequence identity (90% coverage) were clustered using CD‐HIT v4.6.8 software (Fu et al., 2012), and representative sequences containing the longest sequences from each cluster were used to construct non‐redundant gene catalogs. The gene abundance of the metagenomics data was evaluated and quantified with Salmon (Patro et al., 2017). The abundance information of each gene in each sample was calculated from the number of reads and gene length (Villar et al., 2015).

### 2.6 Species annotation

For each sample, taxonomy annotation was done using the Kraken2 v2.1.2 software package with MiniKraken2_v2 database (Kubiritova & Gardlik, 2019; Wood, 2014). The number of genes supporting reads in each species and each family were merged by the MetaPhlAn2 to obtain the final gene catalogue for subsequent analysis (Duy et al., 2015). Multiple alignment results for each sequence arose after filtering, yielding different species classification information. Thus, to ensure its biological significance, the alignment was conducted using mpa format for each sequence alignment for subsequent analysis. Before the cluster analysis, we used the Python script to merge the gut microbial abundance of these seven species. Based on the annotation results and the gene abundance table, the abundance information of each sample at each classification level was obtained. The cluster analysis selected the top 35 genera of the relative abundance table for visualization. The abundance of a species was determined as the average of the gene abundance of the three samples. For each sample, the abundance of gut microbiota in a sample was equal to the number of reads with abundances greater than 0. Therefore, the relative abundance of gut bacterial at the family and genus taxonomical levels was conducted.

### 2.7 Bioinformatics and statistical analysis

Based on the relative abundance table of different classification levels, principal co‐ordinates analysis (PCoA) was conducted with vegan packages and anosim() function, non‐metric multidimensional scaling (NMDS) was conducted with vegan packages and metaMDS() function, LDA effect size analysis (LefSe) was conducted with the MASS, and ggplot2 packages in R (https://www.R‐project.org/) to analyze the main distribution characteristics and the similarity of community samples.

### 2.8 Functional database and resistance gene annotation

Several methods were used for function annotation in this study. First, functional annotation of metagenomes was conducted using the online KEGG automatic annotation server (KAAS; http://www.genome.jp/kegg/kaas/) to blast unigenes to KEGG database (Moriya et al., 2007). Gene ontology (GO) annotation of all the unigenes was performed by the hypergeometric distribution algorithm based on molecular function, biological process, and cellular component. Converted KEGG Orthology (KO) was compared among the groups. After calculating, integrating, and standardizing the abundance of each category in the KEGG pathway, we used the ggplot2 package in R to draw a stacked histogram. Next, we used stamp v2.1.3 software for KEGG differential analysis (Parks et al., 2014).

To further understand the carbohydrate enzymes presented in the gut microbiome, we submitted the samples to the Carbohydrate‐Active enZYmes database (CAZy, Lombard et al., 2014). DIAMOND v0.9.31 software (Buchfink et al., 2021) was used to compare unigenes with each functional database (blastp, evalue ≤1e−5). Searching the unique genes against the CAZy database (Carbohydrate‐Active Enzymes database), the number of genes corresponding to the six categories of carbohydrate enzymes was obtained. Based on the results of the functional annotations and the gene abundance table, the number of genes in each sample at six CAZy classification levels was obtained. The number of genes with a certain function in a sample was calculated as the number of genes with non‐zero abundance. Based on the abundance table at each classification level, analyses of the number and relative abundance of annotated genes were conducted.

Resistance Gene Identifier (RGI v5.2.0) software provided by CARD was employed to compare unigenes with the CARD database (RGI built‐in blastp, evalue ≤1e−30) (Qin et al., 2010). Based on the comparison results of RGI and the abundance information of unigenes, the relative abundance of antibiotic resistance ontologies (ARO) was calculated. Employing the ARO abundance data, a heat map of abundance distribution was constructed, and ARO differences between groups were analyzed by ANOVA, using aov() and TukeyHSD() functions in R.

## 3 RESULTS

### 3.1 General characteristics of the metagenomic datasets

The output data encompassed a total of 290.82 Gb raw data, with an average of 13.85 Gb per sample. After size filtering and quality control, a total of 286.52 Gb clean data was obtained, which accounted for more than 98% of the raw data, showing that the data met the quality requirements for subsequent analysis. The *de novo* assembly of these clean reads resulted in a total of 24.60 Gb of scaftigs, and a total of 28.10 kb of N50 (Table 1). Based on these scaftigs, unigenes with an average length of 333.92 bp and an average GC content of 49.86% were obtained. These unigenes were then used for taxonomic analysis and functional annotation, and the results of which were summarized in Table 1.

**TABLE 1:** Summary of the 21 metagenomes, including sequencing data, assembling data, and predicted unigenes data in each sample

Family	Species	Sample ID	Raw Base (Gb)	Clean Base (Gb)	Total length of Scaftig (Gb)	Number of Scaftig	Average length of Scaftig (Kb)	N50 length (Kb)	Maximum length of Scaftig (Kb)	Total length of unigenes (Gb)	Number of unigenes	Average length of unigenes (bp)	GC content (%)
Muridae		20043	13.70	13.52	1.74	1,897,056	0.92	1.06	744.37	0.54	2,003,077	271.97	49.66
	*Apodemus nigrus*	20080	13.32	13.11	1.59	1,772,606	0.90	1.03	781.56	0.50	1,886,427	263.20	49.26
		20089	13.70	13.63	0.87	983,703	0.88	1.00	316.11	0.65	1,373,787	472.71	52.19
		20103	13.36	12.91	1.46	1,525,941	0.96	1.17	332.66	0.44	1,634,879	266.64	45.64
	*Micromys minutus*	20104	13.83	13.36	1.69	1,674,341	1.01	1.25	277.38	0.46	1,779,534	256.84	44.27
		20157	14.52	14.14	1.87	1,609,505	1.16	1.50	137.48	0.44	1,824,698	243.40	44.39
		20007	13.77	13.69	0.58	681,840	0.85	0.96	558.86	0.43	927,010	462.40	47.97
	*Niviventer confucianus*	20032	15.03	14.95	0.73	657,223	1.11	1.83	672.07	0.53	1,007,132	529.63	51.76
		20006	16.20	16.04	1.41	1,660,439	0.85	0.95	392.73	0.51	1,717,887	294.24	51.91
		20145	13.00	12.88	0.87	1,086,547	0.80	0.85	569.62	0.48	1,246,745	385.01	51.15
	*Niviventer fulvescens*	20163	13.92	13.81	0.87	901,003	0.97	1.19	674.76	0.64	1,282,297	500.44	52.14
		20180	12.70	12.58	0.73	834,012	0.87	0.96	416.79	0.48	1,075,788	447.36	51.21
		20012	13.71	13.61	0.70	483,999	1.44	2.68	389.69	0.62	993,699	623.38	55.25
	*Rattus tanezumi*	20013	14.66	14.46	0.44	337,760	1.30	2.29	554.14	0.35	602,873	577.49	51.47
		20014	13.37	13.21	0.42	340,515	1.23	1.83	314.18	0.34	603,331	569.63	52.92
Soricidea		20025	12.58	12.27	1.34	1,537,066	0.87	1.00	1422.77	0.38	1,553,980	246.15	47.43
	*Anourosorex squamipes*	20135	12.05	11.74	1.30	1,504,539	0.86	1.00	91.62	0.37	1,570,180	236.48	49.45
		20141	14.82	14.52	1.19	1,478,011	0.80	0.90	581.78	0.38	1,457,195	262.18	45.44
		20024	13.46	13.30	1.64	1,361,487	1.20	1.50	791.65	0.42	1,604,834	262.48	52.54
	*Blarinella griselda*	20140	15.42	15.23	1.81	1,194,414	1.51	2.08	172.67	0.44	1,667,271	263.84	50.87
		20148	13.70	13.56	1.37	1,462,209	0.94	1.08	382.44	0.36	1,430,895	250.39	50.21

### 3.2 Characteristics of the gut microbial diversity of these sympatric species

**FIGURE 1:**
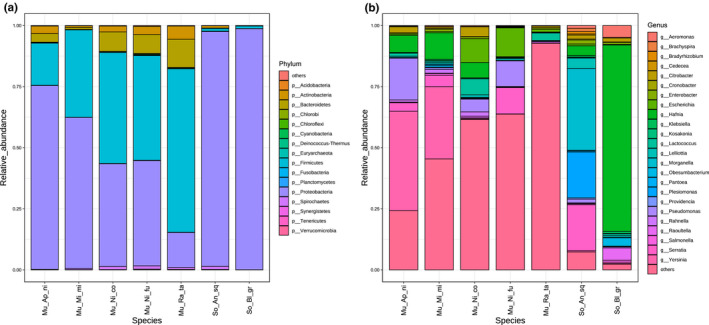
Taxonomic profiles of the microbial communities at the (a) phylum level and (b) genus level in each sample. Sequences that could not be shown into any known groups and that were detected with low abundance were grouped as "others"

**FIGURE 2:**
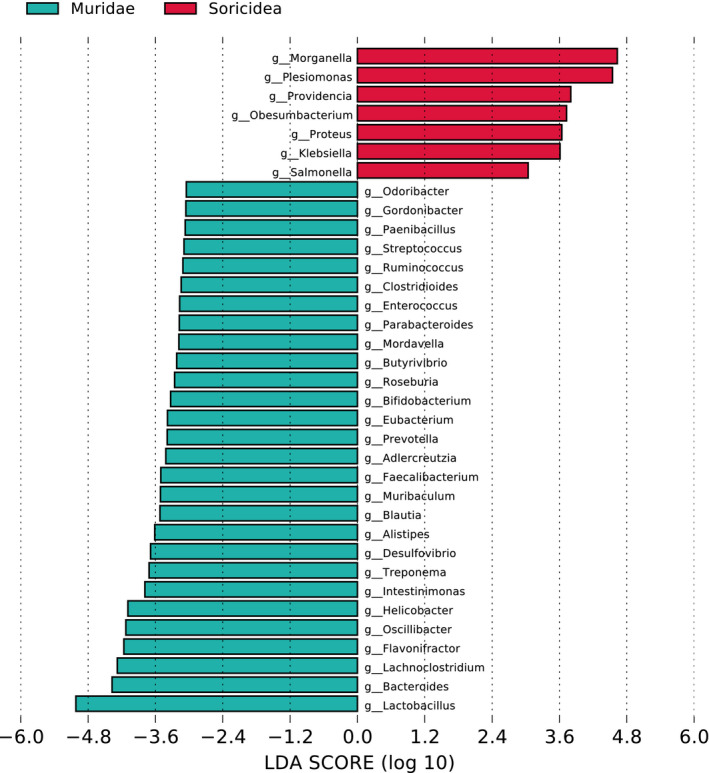
LDA distribution histogram at genus level. The different colored bands represented that the gut microbiota had a difference between the Muridae group and Soricidea group

**FIGURE 3:**
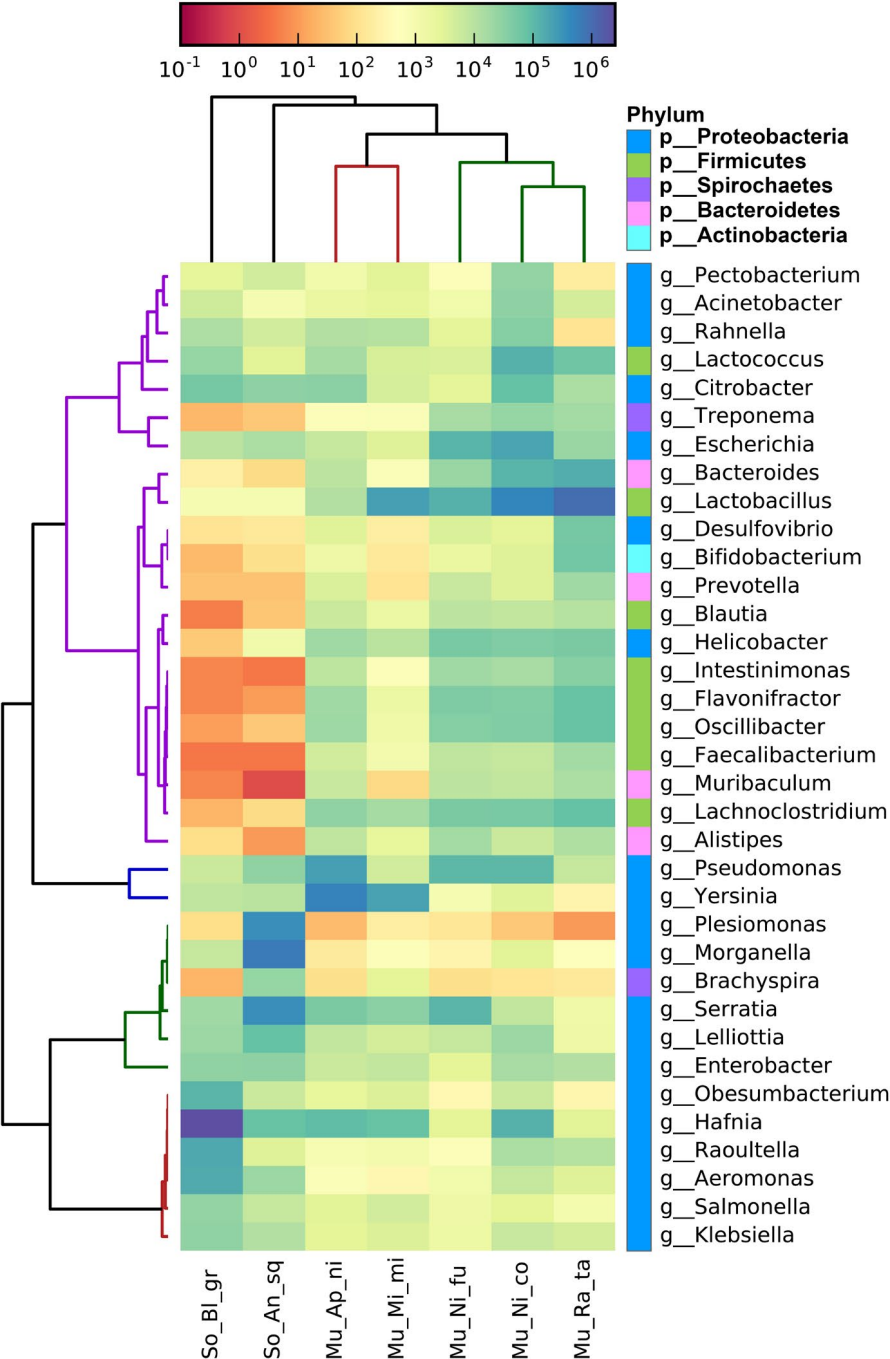
Cluster heat map of relative abundance at genus level. The color of the bar is mapped to the abundance of genera in gut microbes. The positive value is high abundance, and the negative value is low abundance

A total of 2897 classification levels were shared by these seven species and 3297 classification levels were shared by the group Muridae and Soricidea (Figure S1). Based on the relative abundance table of different classification levels, the top 16 phyla and 24 genera and their abundance information were selected to construct a stacked column (Figure 1, Figure S4). Bacteroidetes, Firmicutes, and Proteobacteria were the most abundant phyla in these seven sympatric species. In the Muridae group, the top two Bacteria phyla were Firmicutes (45.52%) and Proteobacteria (41.73%), and the most abundant genus was *Lactobacillus*, followed by *Yersinia*. In the Soricidea group, Proteobacteria also held the overwhelming predominance in Bacteria phyla, with the relative abundance of 97.74%, and the most abundant genus was *Hafnia*, followed by *Morganella*. Difference analysis showed that there were 498 and 122 unique taxonomy levels of gut microbiota in group Muridae and Soricidea, respectively (Table S3). The F/B (Firmicutes/Bacteroidetes) ratio in Muridae group had an advantage over the Soricidea group in the gut microbiota. The F/B (Firmicutes/Bacteroidetes) ratio in the Murine group was 12.64, which was twice than the F/B ratio (6.02) of the Soricidea group (Table S4). Specifically, in Figure 2, the top largest difference genera in abundance of gut microbes between the Muridae and Soricidea were *Lactobacillus*, *Morganella*, *Plesiomonas*, *Bacteroides*, and *Lachnoclostridium*. The genus *Lactobacillus* and *Morgannella* were significantly reversed by these two groups. The number of over 2 showed that there was a significant difference (Guo et al., 2020). In the cluster comparison, the five species of the Muridae clustered together, and the two species of Soricidea located outside of Muridae (Figure 3). Many genera had different proportions in the Muridae and Soricidea.

**FIGURE 4:**
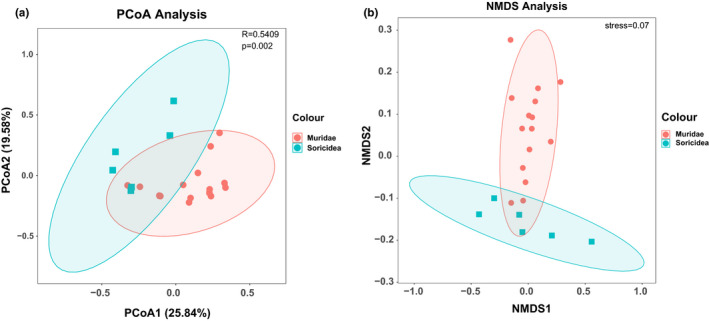
Statistical analysis of data in this study. (a) PCoA plot indicating the microbial phyla distribution between the two groups. (b) NMDS plot indicating the microbial phyla distribution between the two groups

Pairwise binary Bray–Curtis dissimilarities reflected the similarity between communities in terms of the presence/absence of bacterial phylotypes. Because of the complexity of sample data, differences in bacterial community composition between these two groups were estimated using principal coordinates analysis (PCoA) (Figure 4a) and non‐metric multidimensional scaling (NMDS) (Figure 4b) to reduce and simplify the sample data. The results of PCoA showed that there were significant differences between Muridae and Soricidea, and the difference between groups was greater than the difference within groups (*R* = 0.5409; *p* = .002). The results of NMDS showed that the map of NMDS was very representative (stress = 0.07).

### 3.3 Alternative pathway of metabolism in microbiota with different species

**FIGURE 5:**
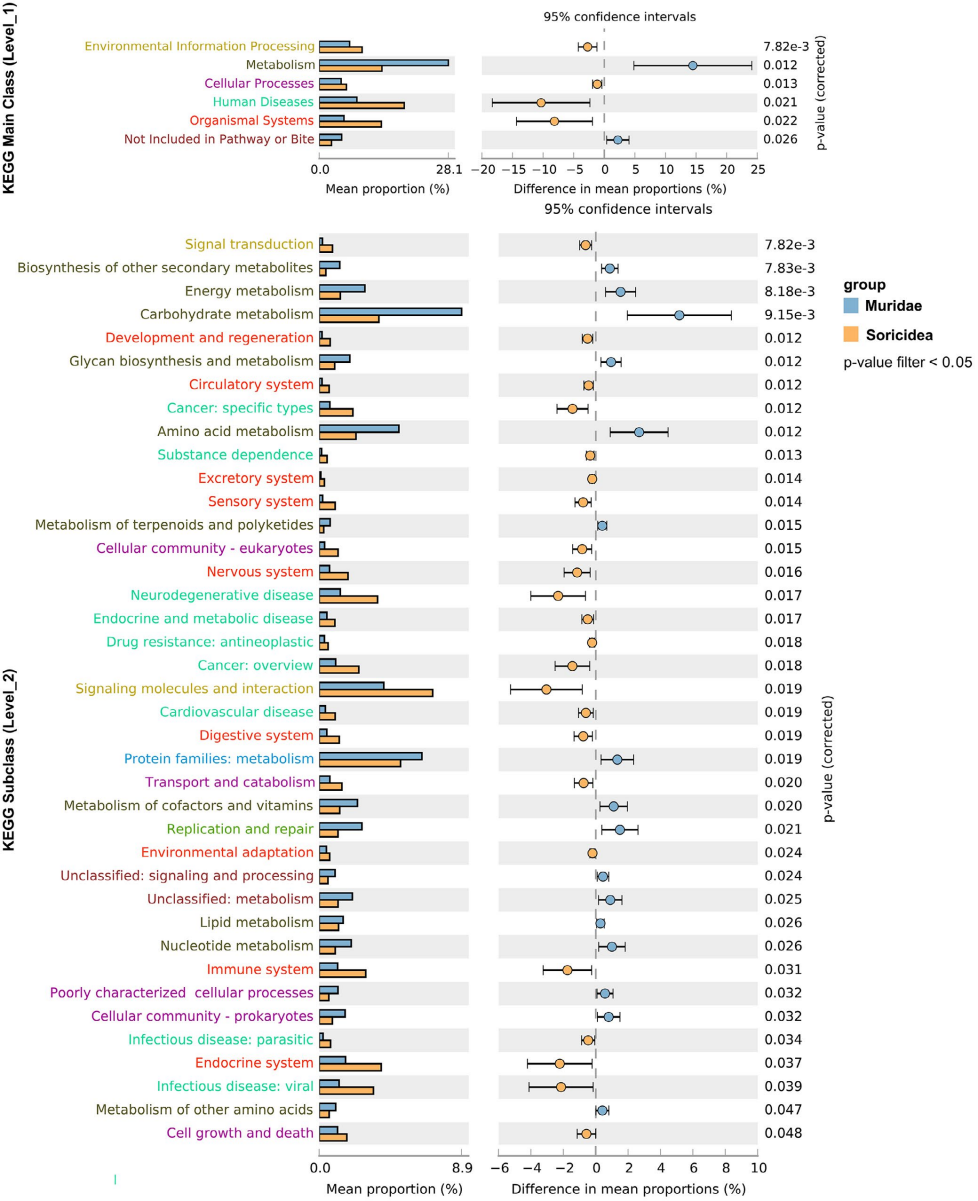
Differential analysis of KEGG pathways in the two groups

The predicted unique genes were searched against the KEGG functional database, and a total of 29,243,519 unigenes were obtained across all samples (Table 2). These unigenes matched to eight level_1 KEGG functional categories, which could be assigned to KEGG ortholog group (KOs) and 344 KEGG pathways (Table 2). KEGG annotation results showed that the genes related to Human disease in *Blarinella griselda* had the highest abundance, while the genes related to Metabolism had highest abundance in other species (Figure S2). Except for Organismal Systems, functions related to Metabolism, Genetic Information Processing, Environmental Information Processing, and Cellular Processes were significantly more abundant in Muridae than that in Soricidea. In the level_2 of category, the genes related to carbohydrate metabolism were the most, which indicated that the metabolic potential of the gut microbiota related to these two groups was highly active. Besides, at the KEGG differential analysis, Metabolism in Muridae had the most obvious difference from that in Soricidea in the level_1 category. And at the level_2 category, Carbohydrate metabolism in Muridae had the most obvious difference from that in Soricidea (Figure 5).

**TABLE 2:** Summary of the number of unigenes used for functional annotation

	Number of matched unigenes	Ratio
Unigenes	29,243,519	–
Functional Annotation		
Annotated on KEGG	6,456,428	22.08%
Annotated on KO	3,686,189	12.61%
Annotated on KO number	214,456 (KOs identified)	–
Annotated on pathway	1,553,040	5.31%
Annotated on pathway number	344 (pathways identified)	–
Annotated on CAZymes	7,671,381	26.23%
Annotated on CARD	46,567	0.16%
Annotated AROs	46163 (AROs identified)	–

### 3.4 Diversity microbial functions in CAZymes and CARD

**FIGURE 6:**
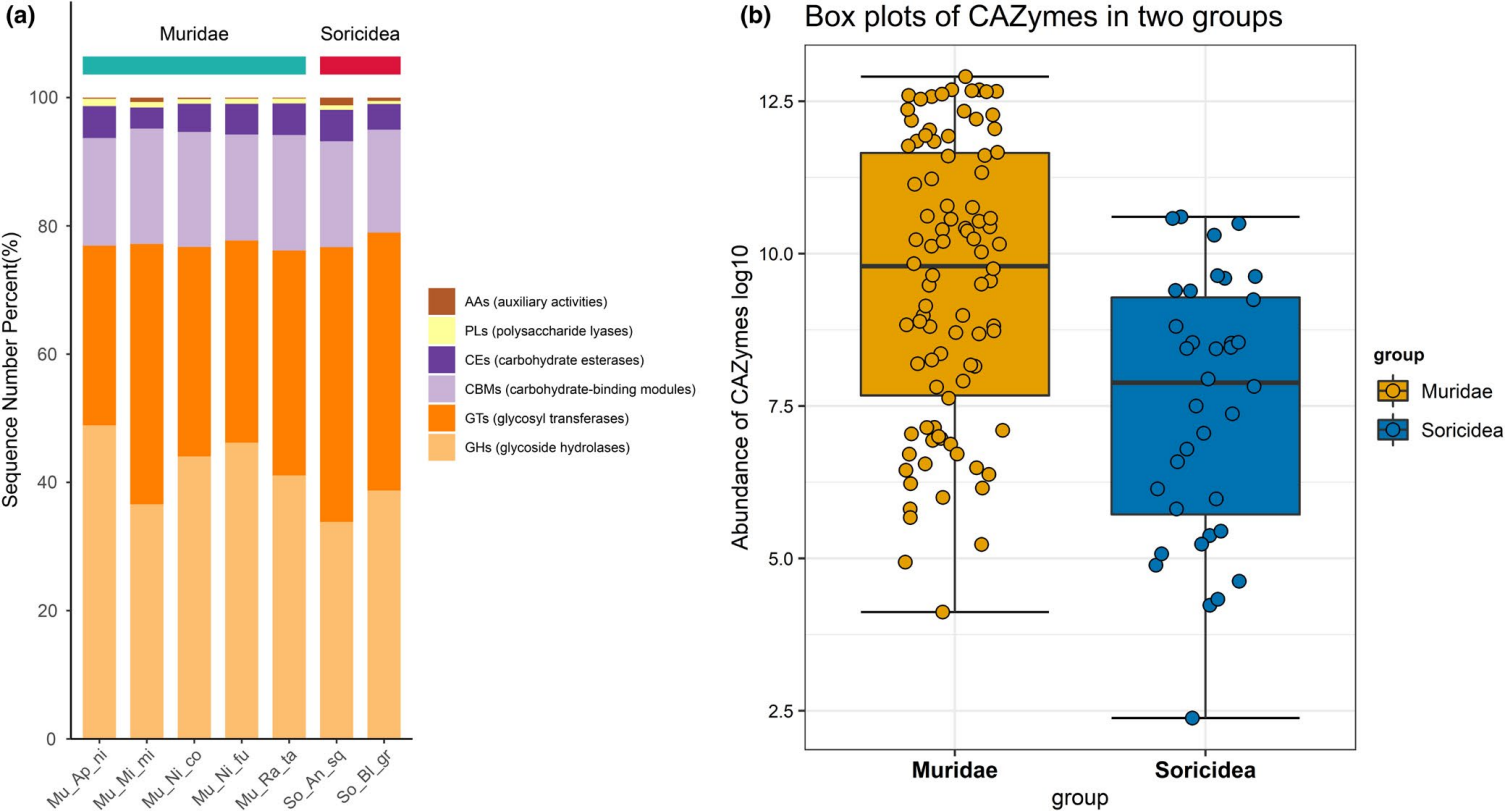
The detected CAZymes in this study. (a) Relative abundance of carbohydrates. (b) Different composition of the six CAZymes categories in gut microbiota of the two groups

A total of 12,950,338 annotation results were obtained for the CAZy database. Based on the annotation results, the relative abundances of genes belonging to the six carbohydrate enzyme categories were plotted in a bar chart (Figure 6a). Among the six largest functional CAZy classes, GHs (glycoside hydrolases) had the highest abundance, reaching 48.52% of the total number of annotation results, followed by GTs (glycosyltransferases, 29.62%) and CBMs (carbohydrate‐binding modules, 16.48%). The difference analysis of CAZymes in gut microbiome showed that CAZymes significantly reduced in the Soricidea group (Figure 6b). In addition, based on the annotation results of the CAZy database, a network diagram of species associated with CAZymes was constructed. In the further functional subclasses, GT2, GT77, and CBM50 were the most three abundant subclasses. At the top 10 functional subclasses, Muridae gut microbiota harbored more CAZymes than that of the Soricidea (Figure S3).

**FIGURE 7:**
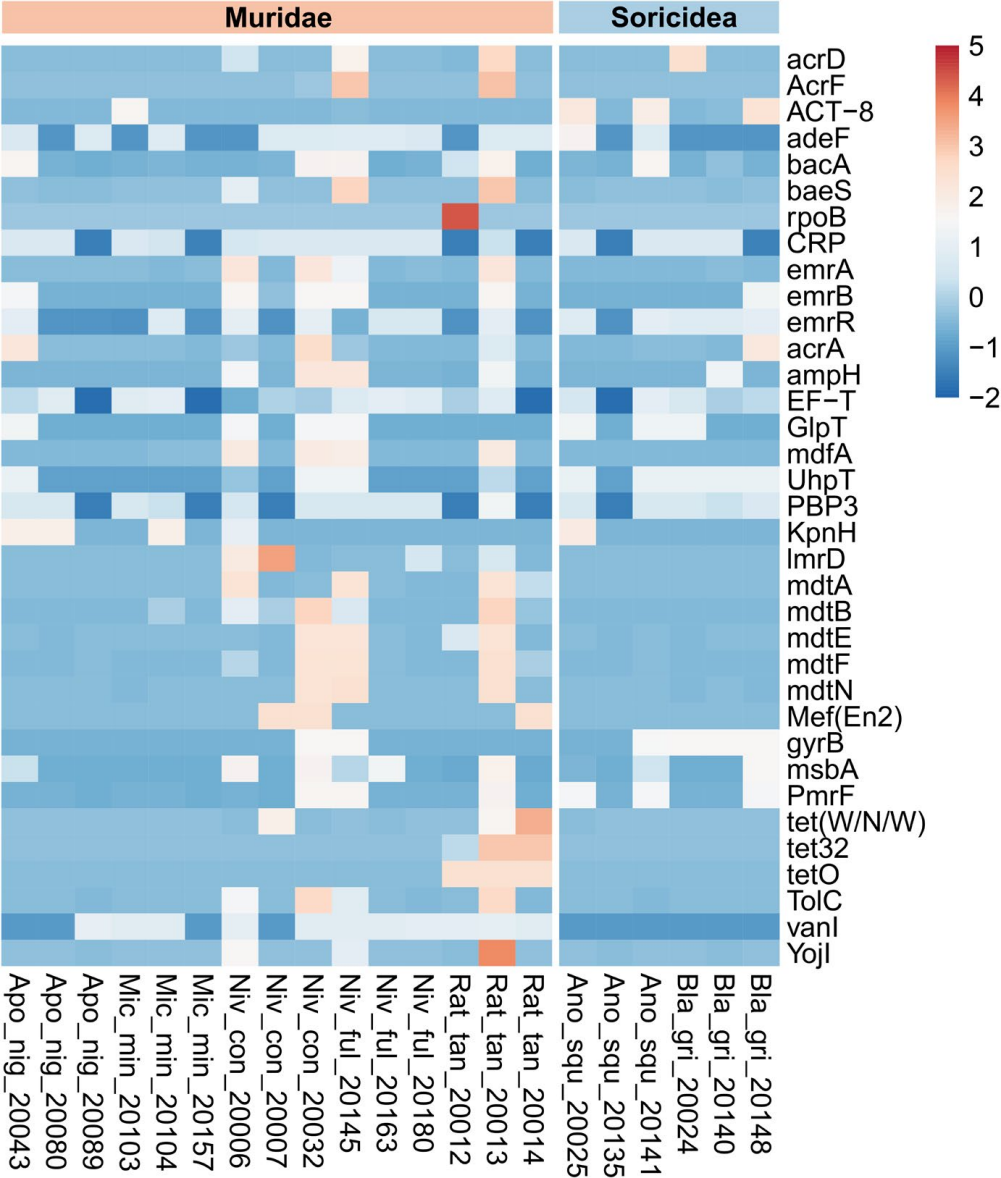
The top 35 AROs distribution and abundance cluster heat map. The right vertical axis is the name of AROs

**FIGURE 8:**
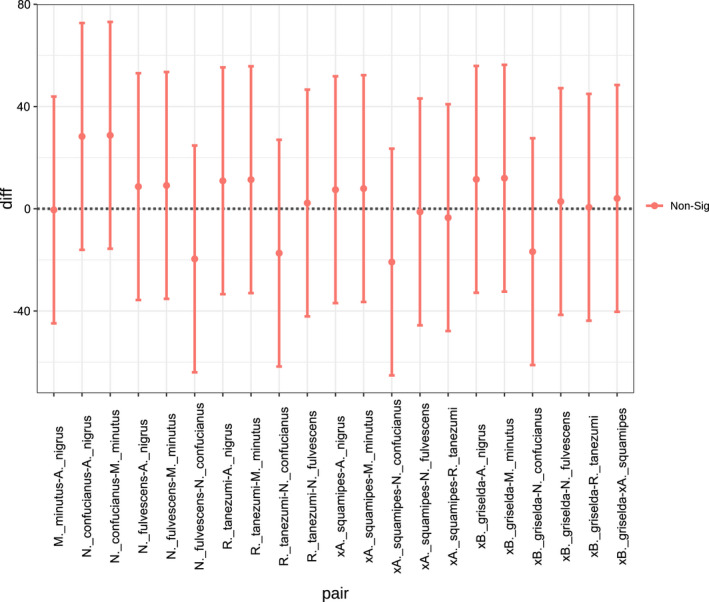
Plot of Tukey's HSD for differences in AROs of seven species

We detected a total of 46,567 ARGs. ARGs presented more abundant in Soricidea than that in Muridae. We found an abundance of multiple drug‐resistant ontologies, in which 46,163 antibiotic resistance ontologies (AROs) were identified and 2,009 AROs were shared by theses seven species, and *N*. *confucianus* displayed high abundance. The top 35 AROs distribution and abundance were displayed in cluster heat map (Figure 7). EF‐Tu, adeF, and PBP3 harbored the biggest difference in the comparison of AROs between these two groups. Regarding the functions of genes in the category ARO gene families, the resistance‐nodulation‐cell division (RND) antibiotic efflux pump was associated with the highest number of genes, accounting for 14.64% of the total ARO genes of all samples. Moreover, the difference analysis of antibiotic resistance genes (ARGs) showed that there was no significant difference in the resistance genes in the gut microbiota of these two groups (ANOVA, *p* = .558, Figure 8).

## 4 DISCUSSION

### 4.1 Convergent gut microbial communities of the seven sympatric species

The gut microbiomes can form a barrier to pathogens and participate in vital physiological and immune processes like maintaining energy homeostasis and metabolism (O'Hara, 2006). The comparison of sympatric species allows us to study the relative influence of genetic and environmental factors on the composition in the gut microbial community of the species (Moeller et al., 2012). Previous researches have shown that the dominant phyla exclusively by species living in sympatry spanned the four major phyla of gut bacteria (i.e., Bacteroidetes, Firmicutes, Proteobacteria, and Actinobacteria) (Huttenhower et al., 2012; Yan et al., 2020; Ye et al., 2016). Proteobacteria and Firmicutes are the most widespread phyla of gut microbiota, and they are commonly observed within gut environments of many mammals, such as human, mouse, and the ruminant animal (Lavrinienko et al., 2018; Maurice et al., 2015). Our results displayed that the major phyla of the seven sympatric species were similar to the previous studies, which were Proteobacteria, Firmicutes, and Bacteroidetes. Seven sympatric shared partially overlapping gut microbial communities spanned the three major phyla and some of the most common taxonomic orders. The environmental factors experienced exclusively by these two sympatric groups had brought the number of bacteria shared by 2897 taxonomy levels, leading to the convergence of the relative abundance of bacterial groups within the gut microbiota (Figure S1). The gut microbiota of Muridae with the Soricidea converged in most of the taxonomic levels. There are several possible explanations for the convergence of the gut microbiota of these seven species, one of which is that the gut bacteria can be shaped by the symbiosis environmental conditions and has shifted between hosts of different species (Campbell et al., 2012; Yatsunenko et al., 2012). For instance, the gut bacteria of unrelated hosts in the same family tend to have similar bacterial sets in human experiments (Yatsunenko et al., 2012); and in mice, hosts kept in the same cage also tend to share gut bacteria (Campbell et al., 2012). Sympatric small mammals may come into contact with each other accidentally, which provides a way for the direct transmission of microorganisms between individuals. Another possible source of the convergence of the gut microbial community in the symbiotic system is dietary overlap and biological characteristics of the hosts. Firstly, host diet can affect the presence or absence and relative abundance of the gut microbiota (Ley et al., 2008). Secondly, sympatric species may develop similar gut environments due to their shared diet, which may favor specific bacterial components (Ley et al., 2008; Moeller et al., 2012).

### 4.2 Dominance of the Bacteroidetes in gut microbiota of the Muridae group compared with the Soricidea group

Our results displayed that the main phyla had different proportions in the two groups, in which Soricidea contained more Proteobacteria and less Firmicutes and Bacteroidetes proportions than Muridae (Table S4). The F/B (Firmicutes/Bacteroidetes) ratio in the Murine group was twice than that in the Soricidea group. In other words, Firmicutes were more dominant in Muridae than the Soricidea group. The F/B ratio may also be considered as a useful obesity biomarker (De Filippo et al., 2010). As members of polysaccharide‐degrading consortia, Firmicutes members are associated with insoluble fiber degradation (Berry, 2016), which contributes to the release of energy from dietary fiber and starch, and they are likely to be a major source of propionate (Thomas et al., 2011). Characterization of less Bacteroidetes has also been reported in some mammalian species, including small mammals and carnivorous species such as bears, cheetahs, giant pandas, red pandas, hedgehogs, and echidnas (Ley et al., 2008). Studies have shown that the presence of few Bacteroidetes was not common, but it may be observed in some carnivorous mammals (Hu et al., 2017; Shinohara et al., 2019). For instance, although giant pandas and red pandas are herbivores, their bodies still retain the characteristics of carnivorous ancestors such as smaller stomachs and alternative digestive tracts (Xue et al., 2015). Therefore, they are not very suitable for digesting bamboo (Dierenfeld et al., 1982; Wei et al., 1999). Moreover, previous studies have shown that the ratio of Firmicutes to Bacteroidetes differs in obese and lean humans, and this proportion decreases with weight loss on low‐calorie diet (Ley et al., 2006). It is therefore reasonable to surmise that the increase in the F/B (Firmicutes/Bacteroides) ratio in the Soricidea group, probably driven by the high‐fat and ‐calorie digestive requirements due to their insectivorous diet. Despite the sympatric distribution, the difference in the abundance of gut microbes displayed the difference in the diet of the host, which may reduce the competitive pressure of the sympatric species, thereby facilitating the coexistence of species (Bagchi & Sankar, 2003).

### 4.3 Species‐specific microbiota and different abundance of CAZymes may enhance the ability of Muridae to extract calories from an herbivorous diet

Gut bacteria play a key role in the digestion of dietary polysaccharides by producing a large number of carbohydrate‐active enzymes (CAZymes) that the host does not produce (Kaoutari et al., 2013). Grains and crops are concentrated sources of dietary fiber, resistant starch, and oligosaccharides (Garron & Henrissat, 2019). Complex carbohydrates are converted into polysaccharides through primary degradation, and then into oligosaccharides (Yadav et al., 2018). The plant‐derived complex carbohydrates are provided by vegetables, cereals, fruits, and leguminous seeds, whereas the animal‐derived dietary glycans are provided by the cartilage and tissue of animals (Koropatkin et al., 2012; Tasse et al., 2010). In this study, the genera *Propionimicrobium*, *Coprothermobacter*, *Acutalibacter*, and *Methanohalobium*, and the species *Candidatus Coxiella* and *Methanothrix* were exclusive to the five species in Muridae group, indicating that the presence of a bacterial community using glucose, lactose, pectin, C5 and C6 sugars, and acetate produced high levels of the complex carbohydrates in the Muridae group (Flint et al., 2008). These bacteria can ferment both xylan and cellulose through carbohydrate‐active enzymes such as xylanase, carboxymethylcellulase, and endoglucanase (http://www.cazy.org). The functional comparation revealed that functions related to carbohydrate metabolism and the abundance of CAZymes were significantly more abundant in Muridae group (Figure 6). Therefore, these results of functional profiling suggested that the gut microbiota of Muridae group often took advantage of these digestible carbohydrates as its main energy source (Gentile & Weir, 2018), and the herbivorous diet in metabolic pathways was beneficial to the use of carbohydrates as fuel to sustain energy expenditure for the Muridae group. These findings were consistent with the fact that the herbivorous Muridae digested plants more easily than the insectivorous Soricidea.

### 4.4 Similar ontologies and concentrations of antimicrobial resistance in the sympatric environment

Highly mobile wild small mammals are exposed to man‐made antibacterial residues, and can acquire, carry, and spread multidrug‐resistant bacteria (Forsberg et al., 2012; Hansen et al., 2016). Based on sequence alignment using a non‐redundant CARD database, we detected resistance genes to commonly used antibiotics in the gut bacteria of all samples and found that these seven species shared all AROs. Previous studies on the impact of agricultural pollution on antimicrobial resistance have shown that interactions with livestock can lead to the spread of antimicrobial resistance to wild animals (Furness et al., 2017). Moreover, wild animals may be exposed to natural antibiotics produced by bacteria and fungi (Poeta et al., 2007). These may be the sources of resistance to these antibiotics in wild small mammals. In addition, there is no significant difference in the ARO carrying rate of the seven species. This may be due to the frequency of antimicrobial resistance in wild animals is similar to that in residential environments (Jardine et al., 2010).

In sum, we present the use of whole‐metagenome shotgun sequencing to analyze the composition and functional structures of the gut microbiota in seven small mammals. Our results displayed that the gut microbial composition and functional structures were consistent with the diet of species. The convergence observed in sympatric species implies that evolutionary differentiation of small mammal gut microbiomes has been maintained by the geographic isolation among host species. We mapped the obtained sequences to genes or pathways in existing databases, such as KEGG, CAZy, and CARD. The functional annotation showed that herbivorous Muridae gut microbiota harbors more CAZymes than that of the insectivorous Soricidea. These enzymes participate in the degradation and modification of carbohydrates and the formation of glycosidic bonds. Furthermore, our results stress the importance of wildlife species as bioindicators for ARO surveillance programs in ecosystems. Comparing sympatric species enabled us to tease apart the influences of heritable and environmental factors on the evolution of small mammal gut microbial communities. These results illustrate the use of different diets to resolve potentially relevant host characteristics, such as geographic and phylogenetic differences, providing nuanced insights into the manner by which gut microbes assort among host species.

## CONFLICT OF INTEREST

We declare that we do not have any commercial or associative interest that represents a conflict of interest in connection with the work submitted.

## AUTHOR CONTRIBUTIONS

**Fengjun Li:** Conceptualization (lead); Data curation (lead); Formal analysis (lead); Investigation (lead); Methodology (lead); Project administration (lead). **Shengzhi Yang:** Methodology (equal); Software (equal). **Linwan Zhang:** Investigation (equal); Methodology (equal); Software (equal). **Lu Qiao:** Formal analysis (equal). **Lei Wang:** Project administration (equal). **Song He:** Data curation (equal). **Jian Li:** Data curation (equal). **Nan Yang:** Data curation (equal). **Bisong Yue:** Conceptualization (equal); Funding acquisition (lead). **Chuang Zhou:** Conceptualization (equal).

## Supporting information

Fig S1

Fig S2

Fig S3

Fig S4

Table S1

Table S2

Table S3

Table S4

## Data Availability

Source and accessibility of the data used in this study have been deposited to Dryad at the following URL: https://doi.org/10.5061/dryad.8cz8w9grk. And the sequencing data have been deposited into CNGBDB at the following URL: https://db.cngb.org/search/project/CNP0002011/
