# Supplementary figures and images for: Comparative metagenomics analysis reveals how the diet shapes the gut microbiota in several small mammals
Source: Ecol Evol. 2022 Jan 15;12(1):e8470. doi: 10.1002/ece3.8470 (PMC8809447; doi:10.1002/ece3.8470)

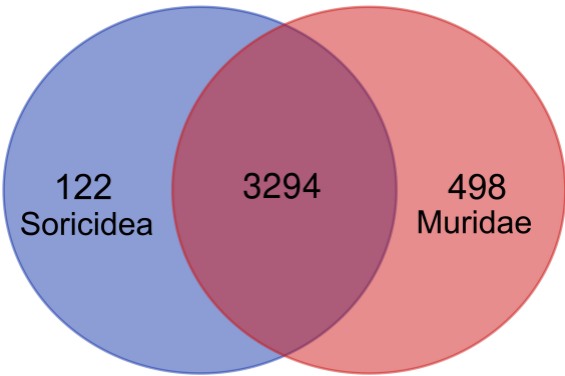

Supplement: Supplementary file 1 — Fig S1 [file ECE3-12-e8470-s004.pdf]

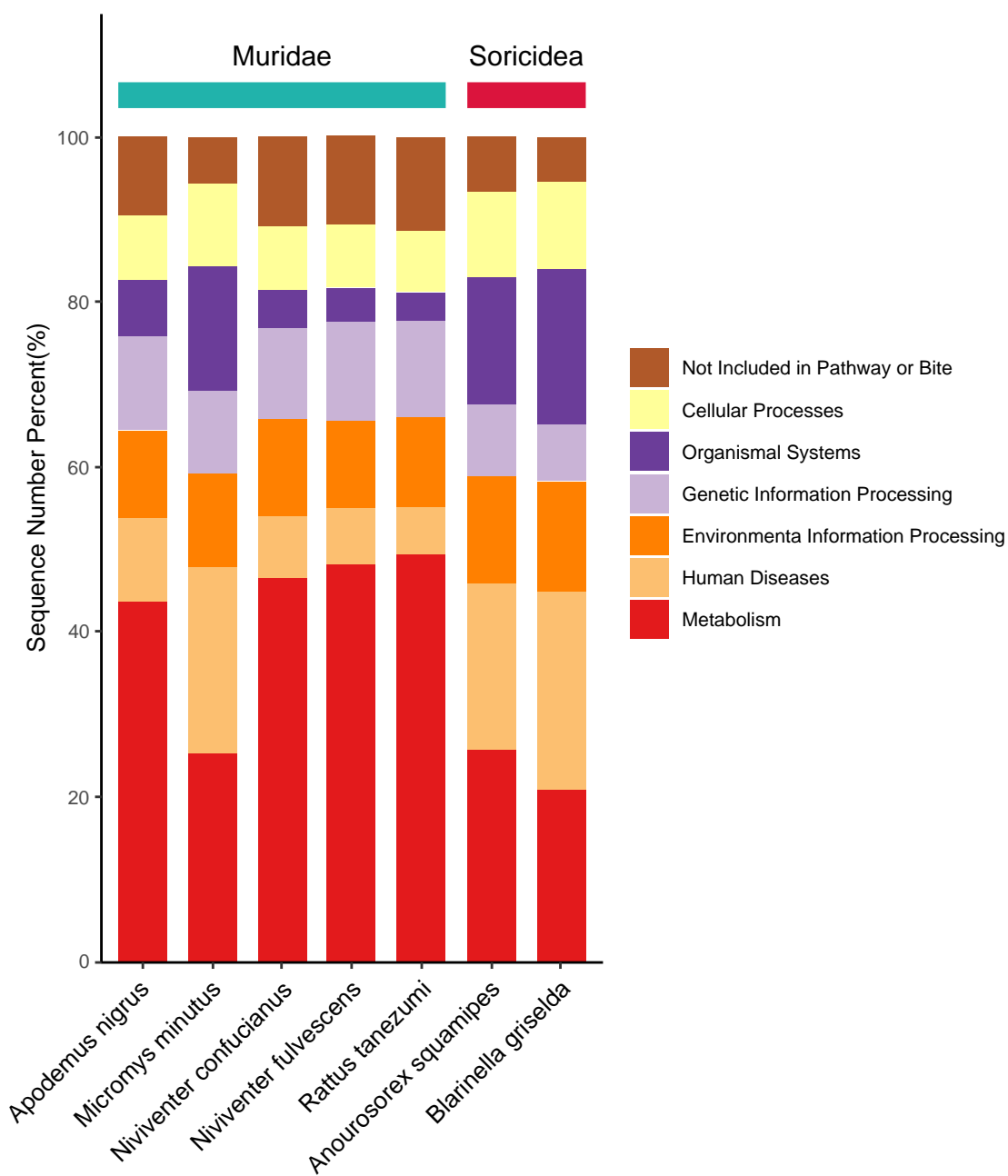

Supplement: Supplementary file 2 — Fig S2 [file ECE3-12-e8470-s005.pdf]

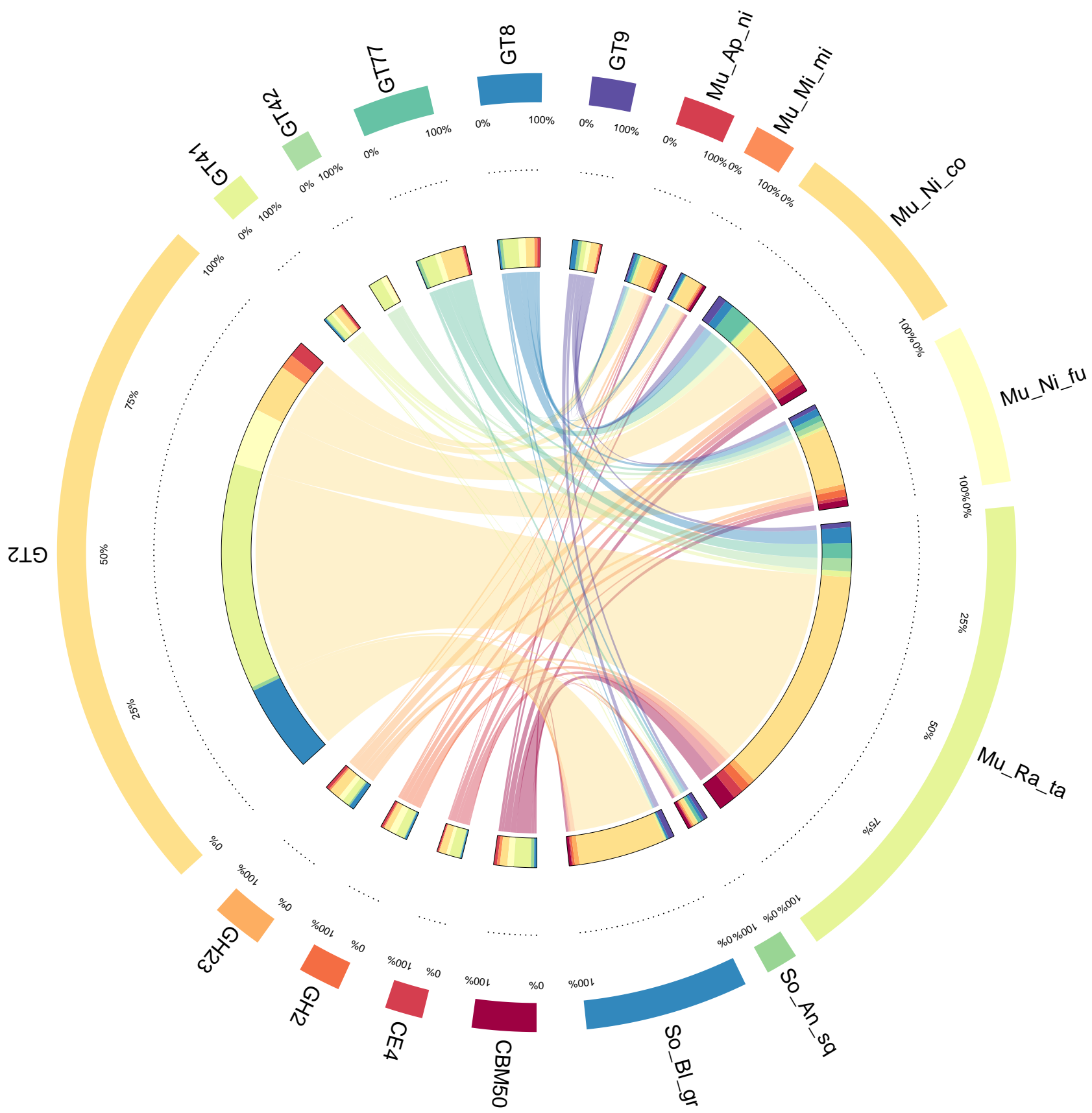

Supplement: Supplementary file 3 — Fig S3 [file ECE3-12-e8470-s008.pdf]

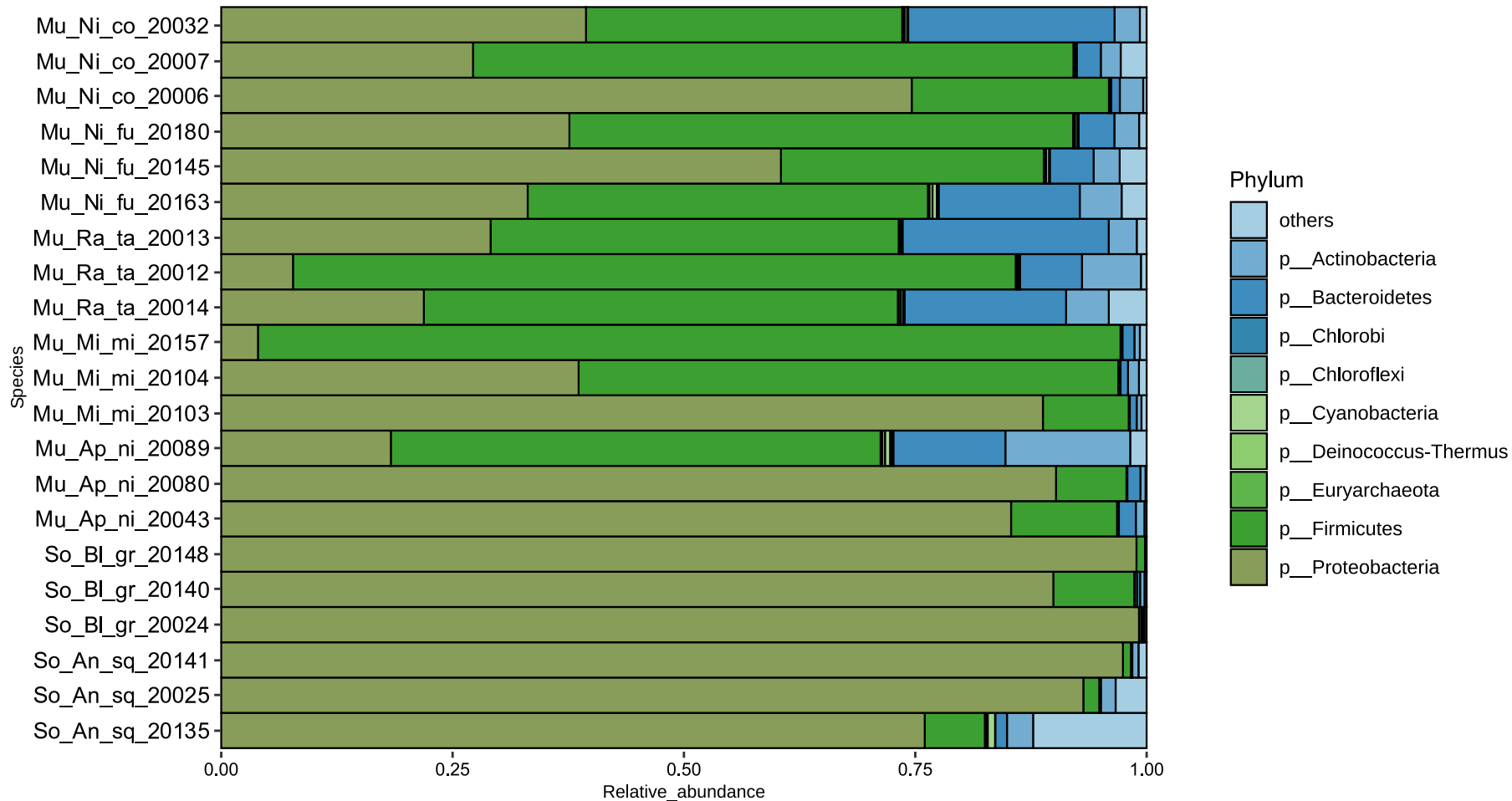

Supplement: Supplementary file 4 — Fig S4 [file ECE3-12-e8470-s002.pdf]
