# Supplementary material for: Comparative metagenomics analysis reveals how the diet shapes the gut microbiota in several small mammals
Source: Ecol Evol. 2022 Jan 15;12(1):e8470. doi: 10.1002/ece3.8470 (PMC8809447; doi:10.1002/ece3.8470)
Supplement: Supplementary file 5 — Table S1 [file ECE3-12-e8470-s003.docx]

TABLE S1. Information of 21 samples of 7 species.

| Family | Species | Sample Number | Altitude (m) | Latitude | Longitude | Collect Date | Sex | Weight (cm) | Body Length (cm) | Tail Length (cm) | Metapedes Length (cm) | Ear height (cm) |
| --- | --- | --- | --- | --- | --- | --- | --- | --- | --- | --- | --- | --- |
| Muridae | *Apodemus nigrus* | 20043 | 1,484 | 28.70 | 104.03 | June 21, 2020 | ♀ | 27.2 | 88.0 | 94.0 | 20.0 | 18.0 |
|  | *Apodemus nigrus* | 20080 | 1,861 | 28.69 | 103.99 | June 21, 2020 | ♀ | 24.9 | 98.0 | 106.0 | 23.0 | 16.0 |
|  | *Apodemus nigrus* | 20089 | 1,861 | 28.70 | 103.99 | June 21, 2020 | ♀ | 27.4 | 105.0 | 34.0 | 14.0 | 10.0 |
|  | *Micromys minutus* | 20103 | 1,861 | 28.70 | 103.99 | June 21, 2020 | ♀ | 11.3 | 70.0 | 74.0 | 15.0 | 10.0 |
|  | *Micromys minutus* | 20104 | 1,861 | 28.70 | 103.99 | June 21, 2020 | ♂ | 10.0 | 66.0 | 70.0 | 14.0 | 11.0 |
|  | *Micromys minutus* | 20157 | 1,535 | 28.70 | 104.03 | June 23, 2020 | ♀ | 12.2 | 62.0 | 78.0 | 15.0 | 10.0 |
|  | *Niviventer confucianus* | 20007 | 1,525 | 28.70 | 104.03 | June 20, 2020 | ♀ | 54.3 | 138.0 | 164.0 | 26.0 | 20.0 |
|  | *Niviventer confucianus* | 20032 | 1,525 | 28.70 | 104.03 | June 20, 2020 | ♀ | 16.5 | 80.0 | 45.0 | 22.0 | 16.5 |
|  | *Niviventer confucianus* | 20006 | 1,525 | 28.70 | 104.03 | June 20, 2020 | ♀ | 51.5 | 127.0 | 172.0 | 24.0 | 22.0 |
|  | *Niviventer fulvescens* | 20145 | 1,493 | 28.70 | 104.22 | June 23, 2020 | ♀ | 44.2 | 119.0 | 157.0 | 27.0 | 19.0 |
|  | *Niviventer fulvescens* | 20163 | 1,460 | 28.60 | 104.02 | June 25, 2020 | ♀ | 60.0 | 140.0 | 170.0 | 28.0 | 20.0 |
|  | *Niviventer fulvescens* | 20180 | 1,499 | 28.70 | 104.04 | June 26, 2020 | ♀ | 106.0 | 145.0 | 182.0 | 28.0 | 18.0 |
|  | *Rattus tanezumi* | 20012 | 1,525 | 28.70 | 104.03 | June 20, 2020 | ♀ | 35.5 | 102.0 | 127.0 | 26.0 | 18.0 |
|  | *Rattus tanezumi* | 20013 | 1,525 | 28.70 | 104.03 | June 20, 2020 | ♀ | 38.5 | 105.0 | 125.0 | 27.0 | 16.5 |
|  | *Rattus tanezumi* | 20014 | 1,525 | 28.70 | 104.03 | June 20, 2020 | ♀ | 36.9 | 105.0 | 130.0 | 26.0 | 18.0 |
| Soricidea | *Anourosorex squamipes* | 20025 | 1,525 | 28.70 | 104.03 | June 21, 2020 | ♀ | 31.2 | 100.0 | 13.0 | 15.0 | / |
|  | *Anourosorex squamipes* | 20135 | 1,537 | 28.70 | 104.03 | June 23, 2020 | ♀ | 21.9 | 90.0 | 15.0 | / | / |
|  | *Anourosorex squamipes* | 20141 | 1,493 | 28.70 | 104.22 | June 23, 2020 | ♀ | 16.1 | 80.0 | 11.0 | 14.5 | / |
|  | *Blarinella griselda* | 20024 | 1,525 | 28.70 | 104.03 | June 21, 2020 | ♀ | 12.7 | 78.0 | 38.0 | 12.0 | 7.0 |
|  | *Blarinella griselda* | 20140 | 1,493 | 28.70 | 104.22 | June 23, 2020 | ♀ | 7.2 | 67.0 | 38.0 | 11.5 | 7.0 |
|  | *Blarinella griselda* | 20148 | 1,513 | 28.70 | 104.03 | June 23, 2020 | ♀ | 9.0 | 65.0 | 34.0 | 12.0 | 6.0 |
