# Supplementary material for: Comparative metagenomics analysis reveals how the diet shapes the gut microbiota in several small mammals
Source: Ecol Evol. 2022 Jan 15;12(1):e8470. doi: 10.1002/ece3.8470 (PMC8809447; doi:10.1002/ece3.8470)
Supplement: Supplementary file 6 — Table S2 [file ECE3-12-e8470-s007.docx]

TABLE S2.The details of data analysis

1. Quality control and genome assembly

bowtie2 -p 12 -x bowtie2_indexes -1 id_1.fq.gz -2 id_2.fq.gz -S id.sam --un-conc id.fq;

megahit -t 24 -m 0.90 --min-contig-len 300 -1 id.1.fq -2 id.2.fq -o id_contig_out;

quast.py id.contigs.fa -o id.megahit.report;

1. Gene prediction and gene abundance analysis

prodigal -p meta -a id_pro.contigs.fasta -m -d id_nuc.contigs.fasta -o id.fna -f gff -s id_poteintial.stat -i id.contigs.fa.gz;

cd-hit-est -i id_nuc.contigs.fasta -o id_nr.fna -M 32000 -T 24 -c 0.95 -aS 0.9 -G 0 -g 1 -d 0;

salmon index -t id_nr.fna -i id_index;

salmon quant -i id_index --libType IU -1 id.1.fq.gz -2 id.2.fq.gz -o id_quant --meta;

1. Species annotation

kraken2 --db ${minikraken2_v2_8GB_201904_UPDATE} --threads 12 --use-mpa --report id_report --paired id.1.fq.gz id.2.fq.gz;

python metaphlan_hclust_heatmap_bug.py -c Spectral --top 35 --minv 0.1 -s log --tax_lev g -x 0.1 -y 0.13 --clust_line_w 1 --in Bacteria7.txt --out id.pdf

1. Functional database and resistance gene annotation

diamond blastx --threads 12 --db CAZy.dmnd --query id_nuc.contigs.fasta --out id_nr.tsv;

rgi main -i id.contigs.fa.gz -o id.rgi -n 12
