# Supplementary material for: Comparative metagenomics analysis reveals how the diet shapes the gut microbiota in several small mammals
Source: Ecol Evol. 2022 Jan 15;12(1):e8470. doi: 10.1002/ece3.8470 (PMC8809447; doi:10.1002/ece3.8470)
Supplement: Supplementary file 7 — Table S3 [file ECE3-12-e8470-s006.docx]

TABLE S3. Uniq gut microbiota in Soricidea and Muridae.

|  | species | genus | family | order | class | phylum | total |
| --- | --- | --- | --- | --- | --- | --- | --- |
| Soricidea | 78 | 34 | 10 | 0 | 0 | 0 | 122 |
| Muridae | 326 | 139 | 29 | 2 | 1 | 1 | 498 |
