# Supplementary material for: Comparative metagenomics analysis reveals how the diet shapes the gut microbiota in several small mammals
Source: Ecol Evol. 2022 Jan 15;12(1):e8470. doi: 10.1002/ece3.8470 (PMC8809447; doi:10.1002/ece3.8470)
Supplement: Supplementary file 8 — Table S4 [file ECE3-12-e8470-s001.docx]

TABLE S4. Shared taxonomic classification of gut microbial communities in Soricidea and Muridae at the phylum level. Abundance is represented by reads.

| Shared phylum levels | Muridae | Soricidea |
| --- | --- | --- |
| Proteobacteria | 692074 | 2693338 |
| Firmicutes | 754787 | 29681 |
| Bacteroidetes | 125310 | 2348 |
| Actinobacteria | 56410 | 10572 |
| Spirochaetes | 13894 | 12709 |
| Cyanobacteria | 2971 | 879 |
| Tenericutes | 806 | 2446 |
| Deinococcus-Thermus | 2095 | 118 |
| Fusobacteria | 654 | 1196 |
| Euryarchaeota | 1027 | 278 |
| Planctomycetes | 778 | 469 |
| Chloroflexi | 1035 | 125 |
| Verrucomicrobia | 1022 | 132 |
| Synergistetes | 1039 | 15 |
| Acidobacteria | 617 | 359 |
| Chlorobi | 836 | 30 |
| Thermotogae | 272 | 80 |
| Fibrobacteres | 285 | 3 |
| Gemmatimonadetes | 210 | 31 |
| Deferribacteres | 194 | 31 |
| Nitrospirae | 165 | 57 |
| Lentisphaerae | 211 | 8 |
| Chlamydiae | 120 | 47 |
| Aquificae | 149 | 18 |
| Chrysiogenetes | 114 | 6 |
| Crenarchaeota | 51 | 54 |
| Ignavibacteriae | 92 | 8 |
| Elusimicrobia | 79 | 11 |
| Candidatus Saccharibacteria | 62 | 17 |
| Armatimonadetes | 68 | 7 |
| Calditrichaeota | 69 | 5 |
| Thermodesulfobacteria | 47 | 7 |
| Kiritimatiellaeota | 44 | 4 |
| Dictyoglomi | 23 | 11 |
| Thaumarchaeota | 18 | 9 |
| Candidatus Cloacimonetes | 12 | 3 |
| Caldiserica | 6 | 4 |
| Candidatus Gracilibacteria | 4 | 1 |
